# Normalization of clonal diversity in gene therapy studies using shape constrained splines

**DOI:** 10.1038/s41598-022-05837-0

**Published:** 2022-03-09

**Authors:** L. Del Core, D. Cesana, P. Gallina, Y. N. Serina Secanechia, L. Rudilosso, E. Montini, E. C. Wit, A. Calabria, M. A. Grzegorczyk

**Affiliations:** 1 University of Groningen - Bernoulli Institute for Mathematics, Computer Science and Artificial Intelligence, Groningen, Netherlands; 2 IRCCS Ospedale San Raffaele, San Raffaele Telethon Institute for Gene Therapy (SR-Tiget), Milan, Italy; 3 Università della Svizzera italiana - Institute of Computing, Lugano, Switzerland

**Keywords:** Statistical methods, Stem-cell research

## Abstract

Viral vectors are used to insert genetic material into semirandom genomic positions of hematopoietic stem cells which, after reinfusion into patients, regenerate the entire hematopoietic system. Hematopoietic cells originating from genetically modified stem cells will harbor insertions in specific genomic positions called integration sites, which represent unique genetic marks of clonal identity. Therefore, the analysis of vector integration sites present in the genomic DNA of circulating cells allows to determine the number of clones in the blood ecosystem. Shannon diversity index is adopted to evaluate the heterogeneity of the transduced population of gene corrected cells. However, this measure can be affected by several technical variables such as the DNA amount used and the sequencing depth of the library analyzed and therefore the comparison across samples may be affected by these confounding factors. We developed an advanced spline-regression approach that leverages on confounding effects to provide a normalized entropy index. Our proposed method was first validated and compared with two state of the art approaches in a specifically designed in vitro assay. Subsequently our approach allowed to observe the expected impact of vector genotoxicity on entropy level decay in an in vivo model of hematopoietic stem cell gene therapy based on tumor prone mice.

## Introduction

Gamma retroviral and Lentiviral Vectors (LVs) are widely adopted in Gene Therapy (GT) thanks to their ability to insert therapeutic transgenes in the host cell genome of hematopoietic stem/progenitor cell (HSPC). After transplantation into the patient the HSPCs reconstitute the entire hematopoietic system and correct the genetic defect. Therefore, vector integration ensures the maintenance of gene correction during self-renewal of HSPCs as well as its transmission to their cell progeny^1^. These vectors integrate semi-randomly within the human genome, and then each transduced cell harbours a vector integration in a distinct genomic position (integration site, IS) that can be adopted as a genetic mark to distinguish each engrafted clone. The retrieval of IS from transduced cells can be done by using PCR protocols that allow to specifically amplify the vector/genome junctions from their genomic DNA. Sequencing and mapping on the target cell reference genome allow to identify IS that can be univocally used for clonal identity. Therefore, the analysis of vector IS from DNA of blood cells harvested at specific time points after transplant from GT patients provide information on number of hundreds to thousands of clones present in circulation and their relative abundance. For this reason, IS studies are required for safety and long-term efficacy assessment in preclinical and clinical studies^2–8^.

The Shannon entropy index, a well-established measure of species diversity in ecology^9^, has become one of the most widely used measure of IS diversity in HSC-GT applications^10^. This measure has been positively correlated to high levels of genetic modification and engraftment of genetically modified cells while low levels of entropy were associated to poor levels of genetic modification, or oligoclonality due to poor engraftment or even the appearance of highly dominant clones resulting from malignant transformation^11^. Indeed, the complexity of a given DNA sample is computed considering both the total number of different IS obtained and their relative abundance. Thus, highly polyclonal samples characterized by large number of IS whose abundance is evenly distributed will have a higher Shannon diversity index than oligoclonal samples with a relatively smaller number of IS and/or characterised by the presence of highly dominant clones. However, the Shannon diversity index does not consider variations in sample size (amounts of DNA analyzed) or the efficiency in species retrieval (different PCR protocols for IS retrieval, sequencing platforms and sequencing depth) of complex ecosystems, such as the population of vector integrations sites in the genome^12^.

Thus, while Shannon diversity index provides an objective measure of the clonal complexity of any given IS sample, these confounding factors should be taken in account when the clonal complexity of different samples is compared. Since longitudinal studies of GT patients for IS monitoring could require the analysis of several samples collected under heterogeneous technical conditions, a method aimed at removing confounding effects in diversity index is needed. To remove confounding factors in the estimations of ecosystem diversity, several methods have been applied. Random subsampling without replacement, called “rarefying”^13^, is among the most popular methods for the normalization of species count data in ecology as well as for next generation sequencing (NGS) data in microbiology. Given a predefined sequence depth (total count, SD), a subsample from each library is generated by randomly picking reads without replacement, until the selected total number of counts is reached. Although rarefying has become the state-of-the-art tool for NGS data analysis^14^, some limitations have been recognized. Indeed^15^, demonstrated that rarefying is statistically inadmissible and should be avoided. Furthermore, in^16^ it was highlighted that estimates of species diversity in sites/habitats at local scale, namely the $\alpha$-diversity^17^, for rarefied microbiome count data may be strongly biased. This is mainly due to the rare species which may be over- (or under-) represented in the samples that have been normalized to a smaller depth by rarefaction. An alternative normalization to rarefying is scaling, which adjusts the size of all samples by scaling their counts to the same total amount. Scaling preserves the relative frequencies of the species and keeps the species richness unchanged. Therefore, simple scaling does not remove the effect provided from the library depth neither on species richness nor on species diversity. Beule^18^ introduces a novel normalization method for species count data called scaling with ranked subsampling (SRS) and the authors demonstrate its suitability for the analysis of microbial communities.

The growing number of normalization and scaling approaches highlights that a robust method has not been developed yet. In this work we show that all proposed methods have limitations. In particular they miss of a precise quantification of the effect of each confounding variable on the Shannon entropy. Furthermore, we also show that the rescaled Shannon entropy index obtained by either rarefying or scaling with ranked subsampling still suffers from the effect of the confounders. We propose a spline-regression approach aimed to explain and remove those effects from the diversity indexes. The effect of the confounders is measured using a B-spline term whose shape is restricted according to a biological-sustained hypothesis. We test our framework by analysing a novel in-vitro dataset properly designed to simulate the same clonality state under different combinations of technical conditions. We also compare our method with the previously proposed methods from the literature in terms of efficiency according to hypothesis testing. That is, we consider a rescaling method to be more efficient if there is more evidence for the corresponding rescaled measure being independent from the effect of the candidate confounders. Finally, our rescaling approach allowed to unmask the expected impact of vector genotoxicity on entropy level decay in an in vivo model of hematopoietic stem cell gene therapy based on tumor prone mice^19,20^.

## Next generation sequencing of clonal tracking data from gene therapy studies

There are several high-throughput systems capable to quantitatively track cell types repopulation from an individual stem cell after a gene therapy treatment^21–23^. Tracking cells by random labeling is one of the most sensitive systems^24^. In HSC-GT applications, haematopoietic stem cells (HSCs) are sorted from the bone marrow of the treated subject and uniquely labeled by the random insertion of a viral vector inside its genome. Each label, called clone, or integration site (IS), is defined as the genomic coordinates where the viral vector integrates. After transplantation, all the progeny deriving through cell differentiation inherits the original labels. During follow-up, the labels are collected from tissues and blood samples using Next Generation Sequencing (NGS)^25–28^. NGS is a recent approach for DNA and RNA sequencing, which consists of a complex interplay of chemistry, hardware, optical sensors and software^29–32^. In gene therapy applications NGS does allow identifying, quantifying and tracking clones arising from the same HSC ancestor. Over the past decades, clonal tracking has proven to be a cutting-edge analysis capable to unveil population dynamics and hierarchical relationships in vivo^33–36^. Clonal diversity, measuring how many distinct clones are collected and how they distribute, can address some of these aspects. Loosely speaking, the less distinct clones the lower the clonal diversity and in turn the less the system is being repopulated in that particular cell compartment. Furthermore, under the same number of different clones collected, the more their distribution is far from the uniform, the lower the clonal diversity and the more the dominance of few clones, thus suggesting the possible occurrence of an adverse event. The Shannon entropy index^37^, a well-established measure of population diversity in ecology studies^9^, nowadays is hugely used as a proxy of clonal diversity in gene therapy applications^10^. Following^37^, the Shannon entropy index is defined as

1 $$\begin{aligned} h(X) = -\sum _{i = 1}^{n}P(x_i)\log P(x_i) \end{aligned}$$

where *X* has possible realisations $x_1,\dots , x_n$ which occur with probabilities $P(x_1),\dots ,P(x_n)$. Therefore, Shannon entropy is a special case (up to a change in sign and a multiplicative factor 1/*n*) of the Kullback-Leibler divergence^38^

2 $$\begin{aligned} D_{KL}(P\Vert Q) = \sum _{i = 1}^{n}P(x_i)\log\frac{P(x_i)}{Q(x_i)} \end{aligned}$$

when the reference *Q* is the uniform distribution on $x_1,\dots , x_n$.

Potential limitations of entropy-based measures in gene therapy applications are related to the heterogeneous nature of NGS data^39–43^. Indeed due to sampling and technical conditions, such as the amount of the host DNA being sequenced and the PCR being adopted, the number of reads obtained per library can span orders of magnitudes^12^ which may affect the cellular counts and in turn their Shannon entropy. These differences in magnitude of library size/depth mainly depends on unequal pooling of PCR products before sequencing. In order to pool PCR products from individual samples in equimolar amounts^44^, DNA concentrations are commonly determined by ultraviolet-visible (UV) or fluorescence spectroscopy, real-time PCR or digital PCR^45^. Although these methods are very effective^46^, an identical library size across samples is difficult to achieve. Nonetheless, if we define the multiplicity of infection (MOI) as the average ratio between the number of virus particles and the number of target cells present in a defined space, then the actual number of viruses that will integrate on any given cell can be described by a stochastic process, such as some cells may absorb more than one infectious agent while others may not absorb any of them. Typically, the probability $P(n\vert m)$ that a cell will absorb *n* virus particles when inoculated with an MOI of *m* can be modelled as a Poisson variable with rate m,

$$\begin{aligned} P(n\vert m) = \frac{m^ne^{-m}}{n!} \end{aligned}$$

Therefore, by definition, it is possible to increase the expected number of vector copies per cell (VCN) by properly tuning the MOI in the design of the experiment/treatment. As a result, the VCN may affect the number of IS collected and in turn the Shannon entropy.

**Figure 1:**
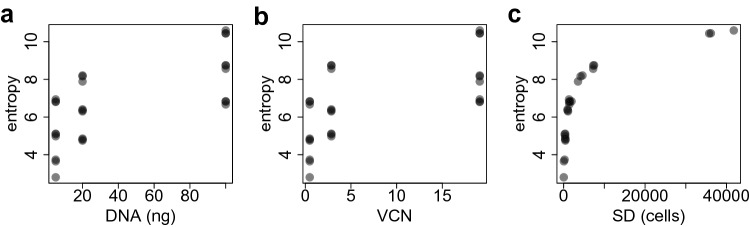
From left to right: Scatter plot of the Shannon entropy index against the DNA amount, the vector copy number (VCN) and the sequencing depth (SD) for all the samples included in the in-vitro assay. Only a single amount of DNA has been taken for every sample. The total amount of integrations found in a sample, namely the total number of sample’s sequencing reads, has been used as proxy for the sample’s sequencing depth (SD).

In Fig. 1 we show the behaviour of the Shannon entropy index as a function of the DNA amount, the VCN and the sequencing depth (SD) in the case of an in-vitro assay described in “In-vitro assay” Section.

Figure 1 suggests that the Shannon entropy index strongly depends on the quantitative confounders, until it reaches a steady-state. These features motivate us to use shape constrained splines (SCS) in order to model the effect of the candidate confounders on the entropy measurements.

## Methods: shape constrained splines

### Definition of the model

Shape-constrained splines (SCS) for fitting, smoothing and interpolation have been explored and proposed in various works, such as^47–52^. In this work we follow the cone-projection approach^51,52^. We model the logarithmic observed entropies $h_i$’s, for $i = 1,\dots , n$, as a function of a SCS-bases ${\mathbf {C}}^k_i$ for every potential confounder $k=1,\dots ,K$ plus a term ${\mathbf {F}}^j_i$ for any other additional feature of interest $j=1,\dots ,J$, so that

3 $$\begin{aligned} \log(h_i) = \beta _0 + \sum _{k=1}^K{\mathbf {C}}^k_{i}\varvec{\beta }_c^k + \sum _{j=1}^J{\mathbf {F}}^j_{i}\varvec{\beta }_f^j + \varepsilon _i \qquad i = 1,\dots , n \end{aligned}$$

where $\beta _0$ is the intercept, ${\mathbf {C}}^k_{i}$ is the basis of a quadratic spline for the *k*-th confounder used for observation *i* for which we assume a saturation state at the right boundary knot and a monotone increasing concave shape. For our applications, the boundary knots of a spline basis associated to a variable *x* are defined as the minimal and maximal value of *x*. The term ${\mathbf {F}}^j_{i}$ corresponds to the *i*-th observation of a basis describing the *j*-th additional component, such as the time or the cell type. The corresponding parameter vectors are $\varvec{\beta }_c^k$ and $\varvec{\beta }_f^j$ respectively. Finally we assume for the noise variable:

$$\begin{aligned} \varepsilon _i \overset{iid}{\sim } N(0,\sigma ^2) \qquad i = 1,\dots , n \end{aligned}$$

Therefore, the model can be compactly written as

4 $$\underbrace {{\log\left( {\left[ {\begin{array}{lll} {h_{1} } \\ \vdots \\ {h_{n} } \\ \end{array} } \right]} \right)}}_{{\log(\mathbf{h})}} = \underbrace {{\left[ {\begin{array}{lll} 1 \\ \vdots \\ 1 \\ \end{array} } \right]}}_{\mathbf{1}}{\beta} _{0} + \underbrace {{\left[ {\begin{array}{lll} {{\mathbf{C}}_{1}^{1} } & \cdots & {{\mathbf{C}}_{1}^{K} } \\ \vdots & {} & {\vdots } \\ {{\mathbf{C}}_{n}^{1} } & \cdots & {{\mathbf{C}}_{n}^{K} } \\ \end{array} } \right]}}_{{\mathbf{C}}}\underbrace {{\left[ {\begin{array}{*{20}c} {{\text{ }}\varvec{\beta} _{c}^{1} } \\ \vdots \\ {{\text{ }}\varvec{\beta} _{c}^{K} } \\ \end{array} } \right]}}_{{\varvec{\beta} _{c} }} + \underbrace {{\left[ {\begin{array}{*{20}c} {{\mathbf{F}}_{1}^{1} } & \cdots & {{\mathbf{F}}_{1}^{J} } \\ \vdots & {} & \vdots \\ {{\mathbf{F}}_{n}^{1} } & \cdots & {{\mathbf{F}}_{n}^{J} } \\ \end{array} } \right]}}_{{\mathbf{F}}}\underbrace {{\left[ {\begin{array}{lll} {{\text{ }}\varvec{\beta} _{f}^{1} } \\ \vdots \\ {{\text{ }}\varvec{\beta} _{f}^{J} } \\ \end{array} } \right]}}_{{\varvec{\beta} _{f} }} + \underbrace {{\left[ {\begin{array}{*{20}c} {\varepsilon _{1} } \\ \vdots \\ {\varepsilon _{n} } \\ \end{array} } \right]}}_{\mathbf{\varepsilon} }$$

where the number of features *K* and *J* depend on the project and/or the specific research questions that must be addressed. Quadratic splines are characterized by the discontinuity of the second-order derivative, which makes their treatments harder than cubic splines. This applies already to unconstrained spline fitting and interpolation. In particular, the definition of quadratic penalised/smoothing splines is not straightforward. Therefore, in general, cubic splines should be preferred over quadratic splines. Despite this, in our in-vitro assay (VA) application only three distinct values of both the DNA amount and the vector copy number (VCN) are available. Therefore, in order to get a good trade-off between bias and variance as well as in order to obtain a full-rank design matrix, we chose quadratic splines with one interior knot for both the DNA amount and the vector copy number (VCN), and a quadratic spline with two interior knots for the sequencing depth. In Sect. S.4.1 of the supplementary material we compare the fits of quadratic and cubic splines (cf. Fig. S.1). For consistency, we also use quadratic splines in the mice study on genotoxicity, where our goal is to evaluate the  impact of the vector design on the entropy decay; cf. Section “Viral vector safety in a mouse genotoxicity study”.

### Shape-constrained splines (SCS) normalization of Shannon entropy

For simplicity, we set $K = 1$ and $J = 0$ in (4), namely we consider only one confounder and no additional factors of interest. The general case can be obtained straightforwardly. Here we follow^53^ and we represent an $(r + 1)$-th order B-spline as

5 $$\begin{aligned} m(x)&= \sum _{j=1}^q\beta _jB_j^r(x) \end{aligned}$$

where, for $j = 1, \dots , q$, the bases are iteratively computed as

6 $$\begin{aligned} B_j^r(x)&= \frac{x - k_j}{k_{j+r+1}-k_j} B_j^{r-1}(x) + \frac{k_{j+r+2} - x}{k_{j+r+2} - k_{j+1}}B_{j+1}^{r-1}(x), \end{aligned}$$

7 $$\begin{aligned} B_j^{-1}(x)&={\left\{ \begin{array}{ll} 1, &{} k_j \le x \le k_{j+1} \\ 0, &{} \text{ otherwise } \end{array}\right. } \end{aligned}$$

for a given sequence of evenly spaced knots $\xi _1 \le \xi _2 \le \dots \le \xi _{q + r + 2}$, where *q* is the number of basis functions and $\beta _j$’s are the corresponding coefficients. The first order derivative of (5) can be written as

8 $$\begin{aligned} m'(x)&= \frac{1}{\delta }\sum _{j=2}^qB_j^{r-1}(x)(\beta _j - \beta _{j-1}) \end{aligned}$$

where $\delta$ is the distance between two adjacent knots. Since all B-spline basis functions are nonnegative by definition, a sufficient condition for $m'(x) \ge 0$, and in turn for the monotone-increasing shape of *m*(*x*), is

9 $$\begin{aligned} \beta _j - \beta _{j-1}&\ge 0 \qquad j = 2, \dots , q \end{aligned}$$

Furthermore, the second order derivative of Eq. (5) can be written as

10 $$\begin{aligned} m''(x)&= \frac{1}{\delta ^2}\sum _{j=3}^qB_j^{r-2}(x)(\beta _j - 2\beta _{j-1} + \beta _{j-2}) \end{aligned}$$

Then a sufficient condition for $m''(x) \le 0$ and in turn for the concavity of the spline in (5) is

11 $$\begin{aligned} \beta _j - 2\beta _{j-1} + \beta _{j-2}&\le 0 \qquad j = 3, \dots , q \end{aligned}$$

The monotonicity and concavity constraints can be written respectively as

12 $$\begin{aligned} V \beta \ge 0 \qquad W\beta \ge 0 \qquad \beta = \begin{bmatrix} \beta _1 \cdots \beta _q \end{bmatrix}' \end{aligned}$$

where

13 $$\begin{aligned} V= \left[ {\begin{matrix} -1 &{} 1 &{} &{} &{} \\ &{} -1 &{} 1&{} &{}\\ &{} &{} \ddots &{} &{} \\ &{} &{} &{} -1 &{} 1 \end{matrix}} \right] \in {\mathbb {R}}^{(q-1) \times q};&& W= \left[ {\begin{matrix} -1 &{} 2 &{} -1 &{} &{} \\ &{} -1 &{} 2 &{} -1 &{} \\ &{} &{} \ddots &{} &{} \\ &{} &{} -1 &{} 2 &{} -1 \end{matrix}} \right] \in {\mathbb {R}}^{(q-2) \times q}; \end{aligned}$$

If both monotonicity and concavity constraints must be satisfied, the first $q - 2$ constraints/rows of $V$ are redundant, as stated by the following Lemma.

#### Lemma 1

*If* $\beta _j - 2\beta _{j-1} + \beta _{j-2} \le 0 \quad \forall j = 3,\dots ,q$* and* $\beta _q - \beta _{q -1} \ge 0$, *then* $\beta _j - \beta _{j-1} \ge 0 \quad \forall j = 2,\dots ,q-1$.

#### Proof


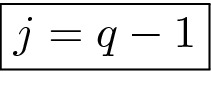
: $\beta _{q} - 2\beta _{q-1} + \beta _{q-2} \le 0$ and $-\beta _{q} +\beta _{q-1} \le 0$ hold, which together imply $\beta _{q-1} - \beta _{q-2} \ge 0$. 
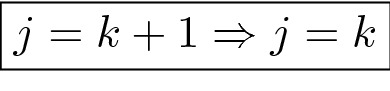
: $\beta _{k+1} - 2\beta _{k} + \beta _{k-1} \le 0$ and $-\beta _{k+1} +\beta _{k} \le 0$ hold, which together imply $\beta _{k} - \beta _{k-1} \ge 0$. $\square$

Therefore by Lemma 1, if both constraints $V$ and $W$ are applied, the whole matrix of constraints reduces to

14 $$\begin{aligned} \quad Z&= \left[ {\begin{matrix} &{} &{} &{} -1 &{} 1 \\ -1 &{} 2 &{} -1 &{} &{} \\ &{} -1 &{} 2 &{} -1 &{} \\ &{} &{} \ddots &{} &{} \\ &{} &{} -1 &{} 2 &{} -1 \end{matrix}} \right] \end{aligned}$$

Furthermore, we need to consider that sampling might be characterised by a sequencing saturation level due to technical limitations. In this case, the saturation level can be included by considering a steady-state/stationary-point at the right boundary knot $\xi _{q + r + 2}$, namely by setting

15 $$\begin{aligned} \partial ^{1} B(\xi _{q + r + 2}) \beta = 0 \end{aligned}$$

where $\partial ^{1} B$ is the first derivative of the spline basis

$$\begin{aligned} B = \begin{bmatrix} B_1^r&\cdots&B_q^r \end{bmatrix} \end{aligned}$$

Our R code implementation allows to switch between the presence and absence of the saturation level by the additional logical input parameter SATURATION. By default this parameter is set to TRUE, but it can be switched to FALSE if the user prefers not to implement a saturation level (or a steady state) w.r.t. a particular predictor variable. In our case of quadratic degree ($r = 1$), the constraint (15) reduces to

16 $$\begin{aligned} \beta _q = -\frac{\partial B[\xi _{q + r + 2}, q - 1]}{\partial B[\xi _{q + r + 2}, q]}\beta _{q-1} \end{aligned}$$

which can be written compactly using the following affine transformation

17 $$\begin{aligned} {\mathcal {A}}: {\mathbb {R}}^{n_X \times q} \rightarrow {\mathbb {R}}^{n_X \times (q - 1)}, X\mapsto XA \end{aligned}$$

18 $$\begin{aligned} A&= \left[ {\begin{matrix} 1 &{} 0 &{} \cdots &{} 0 \\ 0 &{} \ddots &{} \ddots &{} \vdots \\ \vdots &{} \ddots &{} \ddots &{} 0 \\ \vdots &{} &{} 0 &{} 1 \\ 0 &{} \cdots &{} 0 &{} -\frac{\partial B[x_n, q - 1]}{\partial B[x_n, q]} \end{matrix}} \right] \in {\mathbb {R}}^{q \times (q - 1)}; \end{aligned}$$

where $n_X$ is the number of rows of *X*.

### Estimation procedure

Given *n* observations $(x_i, y_i)$ of one predictor *x* and a response *y*, the restricted least squares estimate ${\hat{\varvec{\beta}}}_{RLS}$ of $\varvec{\beta}$ subject to the constraints (14) and (16) can be obtained as

19 $$\begin{aligned} \hat{\varvec{\beta }}_{RLS} = \underset{\varvec{\beta } \in S}{\texttt {argmin}} ({y} - B\varvec{\beta} )'({y} - {B}\varvec{\beta} ) \end{aligned}$$

where

$$\begin{aligned} S&=\left\{\varvec{\beta } \in {\mathbb {R}}^q; \varvec{\beta } \ge 0; Z\varvec{\beta } \ge 0; \beta_q = -\frac{\partial B[\xi _{q + r + 2}, q - 1]}{\partial B[\xi _{q + r + 2}, q]}\beta _{q-1}\right\} \end{aligned}$$

Therefore, using (17)–(18) we can directly include the linear equality constraint

$$\begin{aligned} \beta _q = -\frac{\partial B[\xi _{q + r + 2}, q - 1]}{\partial B[\xi _{q + r + 2}, q]}\beta _{q-1} \end{aligned}$$

inside the objective function, and the optimisation problem (19) reduces to

20 $$\begin{aligned} \hat{\varvec{\beta }}^*_{RLS} = \underset{\varvec{\beta }^* \in S^*}{\texttt {argmin}} \left\{ \underbrace{-2\varvec{\beta }^*BAy + \varvec{\beta }^*(BA)'BA\varvec{\beta }^*}_{f} \right\} \end{aligned}$$

subject to only linear inequality constraints, where

$$\begin{aligned} \varvec{\beta }^* = (\beta _1,\dots ,\beta _{q-1})' \quad \mbox{ and } \quad S^* = \left\{ \varvec{\beta }^* \ge 0; ZA\varvec{\beta }^* \ge 0 \right\} \end{aligned}$$

Since *f* is a quadratic function, we solve (20) using quadratic optimization. To this end we use the function solve.QP() from the R package quadprog. Once the restricted least squares estimate ${\hat{\varvec{\beta}}}^*_{RLS}$ is obtained, we follow the cone projection approach^52^ and we define a point-wise confidence interval (CI) with $1 - \alpha /2$ coverage for $\varvec{b}_p'A\hat{\varvec{\beta }}^*_{RLS}$ as

21 $$\begin{aligned} \varvec{b}_p'A\hat{\varvec{\beta }}^*_{RLS} \mp z_{\alpha /2}\sqrt{{\hat{\sigma }}^2_{RLS}(\varvec{b}_p'A)' \hat{\varvec{G}}\varvec{b}_p'A} \end{aligned}$$

where $\varvec{b}_p = B(x_p)$ is the B-spline basis *B*(*x*) evaluated at the prediction point $x_p$ and the variance is estimated as

$$\begin{aligned} {\hat{\sigma }}^2_{RLS} = \frac{(y - BA\hat{\varvec{\beta }}^*_{RLS})'(y - BA\hat{\varvec{\beta }}^*_{RLS})}{n - 1.5d} \end{aligned}$$

where *d* is the dimension of the cone’s face where the projection of *y* onto

$$\begin{aligned} {\mathcal {F}} = \{ \eta \in {\mathbb {R}}^{n} \vert \eta = BA\varvec{\beta}^* ~\mbox{ such that }~ ZA\varvec{\beta}^* \ge 0 \} \end{aligned}$$

lands on. The dimension *d* of ${\mathcal {F}}$ can be computed using the Hinge algorithm implemented in the R package coneproj^54^. The matrix $\hat{\varvec{G}}$ is computed as the following weighted average

22 $$\begin{aligned} \hat{\varvec{G}} = \sum _{{\mathcal {I}} \subseteq \{1,\dots , q-1 \} } \hat{\varvec{G}}^{({\mathcal {I}})}{\hat{p}}_{\mathcal {I}} \end{aligned}$$

with the $(q-1) \times (q-1)$ matrix $\hat{\varvec{G}}^{({\mathcal {I}})}$ defined as

23 $$\begin{aligned} \begin{aligned} \hat{\varvec{G}}^{({\mathcal {I}})}_{k\in {\mathcal {I}},l \in {\mathcal {I}}}&= ((X_{\mathcal {I}}'X_{\mathcal {I}})^{-1})_{k \in {\mathcal {I}},l \in {\mathcal {I}}} \\ \hat{\varvec{G}}^{({\mathcal {I}})}_{k\notin {\mathcal {I}},l \in {\mathcal {I}}} = \hat{\varvec{G}}^{({\mathcal {I}})}_{k\in {\mathcal {I}},l \notin {\mathcal {I}}} = \hat{\varvec{G}}^{({\mathcal {I}})}_{k\notin {\mathcal {I}},l \notin {\mathcal {I}}}&= 0 \end{aligned} \end{aligned}$$

where $X_{\mathcal {I}}$ are the columns of *BA* indexed by ${\mathcal {I}}$. Each weight ${\hat{p}}_{\mathcal {I}}$ represents the estimated probability that the projection of *y* lands on the cone’s face corresponding to ${\mathcal {I}}$. The probabilities ${\hat{p}}_{\mathcal {I}}$ are obtained by simulating many normal random vectors with mean vector ${\hat{y}} = BA\hat{\varvec{\beta }}^*_{RLS}$ and covariance matrix ${\hat{\sigma }}^2_{RLS}I_n$, and recording the resulting sets ${\mathcal {I}}$’s, along with their frequencies. In case additional unconstrained components are present, the definition of (22) can be extended^52^. Furthermore, if we need to select from a set of candidate models featuring different covariates, we use information criteria^55^. For our analyses we use the corrected Akaike Information Criterion (AICc)

$$\begin{aligned} AIC(M) = -2\log(L(\hat{\varvec{\beta }}^*_{RLS} \vert y)) + 2p + 2p\cdot \frac{p+1}{n - p} \end{aligned}$$

for model selection, where $L(\varvec{\beta } \vert y)$ is the likelihood of model *M* and *p* the number of parameters of *M*, which is equal to *d* in our set-up. In case some models have similar AICc values, we follow Burnham et al.^55^ and we average across all ones using the frequentist model average estimator

24 $$\begin{aligned} \hat{\varvec{\beta}}_{fma}&= \sum _{l=1}^L \lambda _l\hat{\varvec{\beta}}_l \end{aligned}$$

where $\hat{\varvec{\beta }}_l$ is the parameter vector estimated under the *l*-th candidate model, and $\lambda _l$ the corresponding weight which can be computed as

25 $$\begin{aligned} \lambda _l&= \frac{\exp(-BIC_l/2)}{\sum _{j=1}^L \exp(-BIC_j/2)} \end{aligned}$$

where $BIC_j$ is the Bayesian Information Criterion (BIC) associated with the *j*-th model. In case of model averaging, the BIC is preferred over AIC/AICc, since it provides a better estimation of the marginal likelihood^55^. From a Bayesian perspective, $\{ \lambda _j\}_j$ can be interpreted as an estimator of the posterior probabilities

26 $$\begin{aligned} p(M_j | y,X) = \frac{p(y \vert M_j)p(M_j)}{\sum_{l=1}^J p(y \vert M_l)p(M_l)} \qquad j = 1,\dots ,J \end{aligned}$$

of the candidate models under a uniform prior $\{p(M_j)\}_j$, where

27 $$\begin{aligned} p(y | M_j) = \int _{\Theta _j} p(y \vert M_j; \Theta _j)p(\Theta _j \vert M_j) d\Theta _j \end{aligned}$$

is the marginal likelihood^55^.

### Ethics oversight

All procedures were performed according to protocols approved by the Animal Care and Use Committee of the San Raffaele Institute (IACUC 619) and communicated to the Ministry of Health and local authorities according to Italian law. All experiments were performed in accordance with relevant guidelines and regulations. The reporting in the manuscript follows the recommendations in the ARRIVE guidelines.

## Applications of SCS in NGS data

### In-vitro assay

To evaluate the reliability and sensitivity of the SCS rescaling method, we generated an IS dataset originating from an EBV-transformed B cell line transduced with a LV at Multiplicity of Infection (MOI) of 0.1, 1 and 10 to obtain DNA samples with increasing levels of polyclonality. Therefore, by increasing the MOI at each transduction we expect an increase in the vector copy number (VCN). As expected, the different vector doses resulted in different VCNs (see Supplementary Table S.3). Different amounts of DNA (5, 20 and 100 ng) were used for IS retrieval. LV ISs were retrieved by Sonication Linker-mediated (SLiM) - PCR (see Sect. S.1 of the supplementary material). Briefly, DNA material was sheared by sonication, subjected to end-repair and adenylation and then split in 3 technical replicates. Each replicate was ligated to a different barcoded linker cassette and subjected to two rounds of PCR allowing the amplification of the cellular genomic portion close to the vector IS. The different barcoded PCR products from each sample were assembled in libraries and sequenced by using Illumina platform. After sequencing, reads were processed by a dedicated bioinformatic pipeline^56^ to identify for each PCR/sample the different vector integration sites. For each IS the clonal abundance was determined by the R package SonicLength^57^ using the corresponding fragment length distribution. A varying number of ISs, ranging from 22 to 40575, was obtained from each sample (see Supplementary Table S.1) and, as expected, the number of IS retrieved from each sample increased proportionally to the vector dose (see Supplementary Table S.2). The total number of sample’s sequencing reads was used as proxy for the sample’s sequencing depth (SD). The magnitude of VCN, DNA amount and SD affects the clonality so that the samples are incomparable. Indeed Fig. 1 clearly shows a positive trend between the Shannon entropy index and the potential confounders. With the VA we are able to really understand the impact of the variables (confounding factors) to the entropy index, thus allowing a robust integrated analysis. We used the VA as “ground-truth” to compare our SCS-rescaling method with the competitor approaches (RAR and SRS). In our SCS method we took in consideration the DNA amount, VCN and SD as potential confounders.

**Figure 2:**
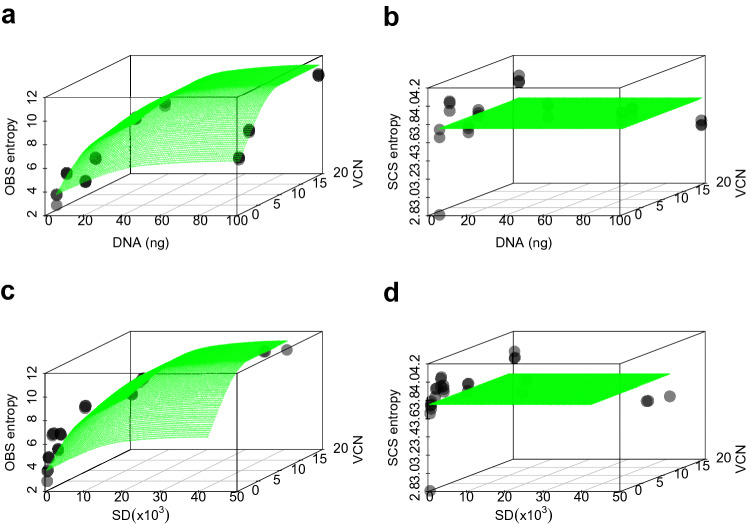
Observed (left) and SCS-rescaled (right) entropies (dot symbols) as a function of the confounders, together with the corresponding shape constrained (bivariate) splines (green surface). Top panels show the slices for the DNA and the VCN. Bottom panels show the slices for the SD and the VCN.

In this case the number of candidate confounders is $K = 3$ with no additional factors of interest ($J = 0$) and, according to the general formulation of Eq. (4), the model was defined as

28 $$\log(\mathbf{h}) = \mathbf{1}{\beta} _{0} + \underbrace {{\left[ {\begin{array}{*{20}c} {{\mathbf{C}}_{{dna}} } & {{\mathbf{C}}_{{vcn}} } & {{\mathbf{C}}_{{sd}} } \\ \end{array} } \right]}}_{{\mathbf{C}}}\underbrace {{\left[ {\begin{array}{*{20}c} {{\text{ }}\varvec{\beta} _{{dna}} } \\ {{\text{ }}{\varvec{\beta}_{vcn}} } \\ {{\text{ }}\varvec{\beta}_{{sd}} } \\ \end{array} } \right]}}_{{\varvec{\beta} _{c} }} + \mathbf{\varepsilon}$$

where we used two equidistant interior knots and the range of values as boundary knots for every SCS term in ${\mathbf {C}}$. We report the corresponding fitted surface in Fig. 2 . In Fig. 2 we also show the rescaled values, i.e. the residuals that remain after having adjusted for the confounders. That is, according to the model definition in Eq. (4), we used the residuals

29 $$\begin{aligned} \mathbf{h}^{res}&= \exp\left( \log(\mathbf{h}) - {\mathbf {C}}\hat{\varvec{\beta }}_c \right) \end{aligned}$$

as the rescaled values, where $\hat{\varvec{\beta }}_c$ is the vector of the fitted parameters.

**Figure 3:**
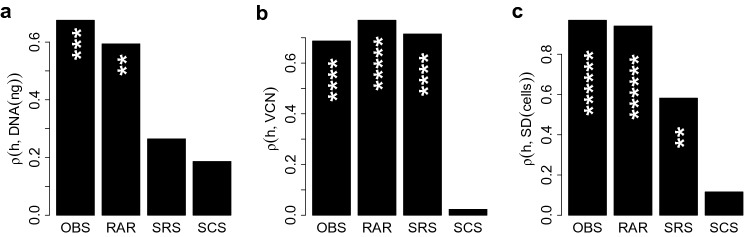
Each panel shows the absolute value of the Spearman’s rank $\rho$ correlation coefficient $\rho (h, \texttt {confounder})$ (*y*-axis) between a confounder and the observed or rescaled Shannon entropies (different bars). For every correlation coefficient, we performed the two-sided Spearman’s rank correlation test of Eq. (30) for checking the hypotheses $H_0 : \rho (h,\texttt {confounder}) = 0 ~~\mbox{ vs }~~ H_1: \rho (h,\texttt {confounder}) \ne 0$. The number of leading zeros after the decimal point of the p-values are reported on top of each bar as white stars.

We compared our method with the two previously proposed in literature, such as the rarefaction (RAR)^13^ and the ranked subsampling (SRS)^18^ approaches. We assessed the efficiency of the rescaling methods by correlation p-values for the two-sided test problem:

30 $$\begin{aligned} H_0 : \rho (X_1,X_2) = 0 ~~\mbox{ vs }~~ H_1: \rho (X_1,X_2) \ne 0 \end{aligned}$$

where $\rho (X_1,X_2)$ is the Spearman’s rank correlation between two random vectors $X_1$ and $X_2$. We preferred Spearman’s rank correlation over Pearson correlation since we assumed that the relationships are monotonic and possibly non-linear. Low *p*-values give statistical evidence for dependencies and thus for unsolved confounding effects. For the comparison, the total amount of reads of the sample with the lowest SD has been chosen as rarefaction level with 1000 replications for both the standard rarefaction (RAR) and its ranked version (SRS). We report the results in Fig. 3. These pictures show that our SCS method outperformed both RAR and SRS methods in terms of correlation test *p*-values between the rescaled entropy and every potential confounder. Indeed, for all three confounders our new approach yields high *p*-values (0.37, 0.15 and 0.31 for DNA, VCN and SD respectively), so that we have no indication to reject the null hypothesis that the rescaled entropies and the confounder values are not correlated. For each of the two competing approaches we got 2-3 very low p-values ($\ll 0.01$), so that statistically significant amounts of correlations are left.

**Figure 4:**
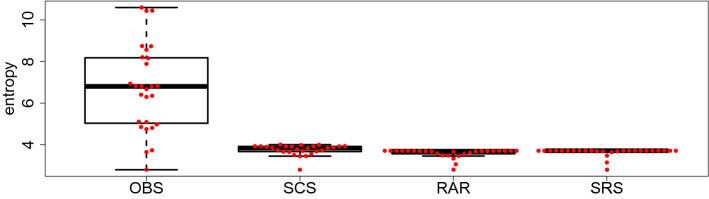
Box-plot (minimum, maximum, median, first quartile and third quartile) of the observed (OBS) and rescaled Shannon entropies using the SCS, RAR and SRS approaches.

Subsequently, we also checked whether our SCS-rescaling method unveils comparable clonal levels among the samples. A proper rescaling method should return similar clonal diversities independently from the confounders. Indeed, Figs. 2 and 4 show that our SCS-rescaling method drastically reduced the variability of the observed entropies due to the effect of the confounders and, in turn, that the clonal level of the VA samples, measured by the SCS-rescaled Shannon entropy index, is approximately the same.

It can be seen from Fig. 4 that the competitor methods RAR and SRS are also able to reduce the variability of the observed entropies. However, unlike our new SCS-rescaling method, the competing methods did not remove the effect of the confounders, as confirmed by the *p*-values of Spearman’s rank correlation tests provided in Fig. 3. While the SCS-rescaling method made all (rank) correlations insignificant, the competing methods RAR and SRS left significant rank correlations (=dependencies) between confounders and the entropy. For more explanations and illustrations we refer to Sect. S.4.2 and Fig. S.2 of the supplementary material.

### Viral vector safety in a mouse genotoxicity study

**Figure 5:**
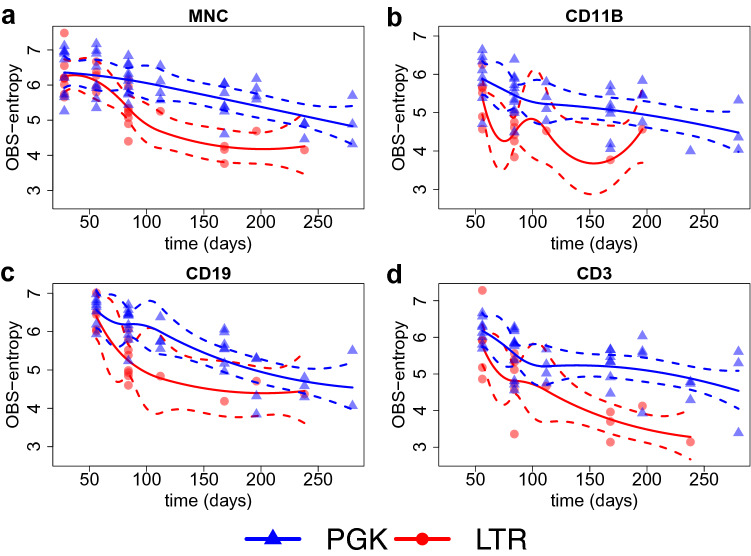
Observed Shannon entropies (*y*-axis) over time (*x*-axis) in each treatment (different colors), along with a simple spline without any shape constraints and confounder adjustments for every combination of cell marker and viral vector. Quadratic splines are fitted using the standard $\texttt {lm()}$ and $\texttt {bs()}$ R functions.

We analysed the IS data collected from an established hematopoietic stem cell gene therapy model previously used to demonstrate how the genotoxic impact of integrating vectors is strongly modulated by their designs^19,20^. In this experimental setup $Cdkn2a^{-/-}$ tumor prone $Lin^-$ cells were ex-vivo transduced with two different LVs expressing GFP: the highly genotoxic LV vector, LV.SF.LTR (hereinafter referred to as LTR) and the non-genotoxic SIN.LV.PGK.GFP.PRE (hereinafter referred to as PGK). Transductions protocol and culture conditions were reported in^19,20^ and further details can be found in Sect. S.3 of the supplementary material. Twenty-four hours after transduction, vector- and mock- transduced cells (5-$7.5\times 10^5$ cells/mouse) were transplanted into lethally irradiated wild-type mice by tail vein injection (Mock-control, N=19; LV.SF.LTR, N=24 and SINLV.PGK, N=23). Six days after transduction the percentages of GFP+ cells were assessed by Fluorescence Activated Cell Sorting (FACS) analysis and ranged from 90 to 95% for the all vector and conditions. Engraftment level of transduced cells was assessed by measuring the percentage of GFP-expressing cells in the peripheral blood at 8 weeks post transplantation and were $80.8 \pm 2.9$ % and $46.2\pm 4.8$ % in the group of mice transplanted with PGK and LTR vector respectively. As expected, mice transplanted with $Cdkn2a^{-/-}$ $Lin^-$ cells transduced with the the LTR vector developed tumors and died significantly earlier compared to mock-treated mice ($p<0.0001$, Log-rank Mantel-Cox test, median survival time: 282 and 149.5 days for mock-control and LTR- transduced group respectively). Mice transplanted with PGK-transduced cells did not show any acceleration of tumor onset compared to the mock-control group (median survival time: 289 days). All data are in agreement with the one previously published^19,20^. For the retrieval of vector insertion sites (ISs), peripheral blood was collected on a monthly basis from transplanted animals receiving transduced cells. Lymphoid B and T cells as well as myeloid cells were isolated by fluorescence activated cell sorting. To recover enough DNA material, equal amounts of blood from two or three mice belonging to the same experimental group were pooled before the sorting procedure. The composition of pools was maintained constant during the whole experiment, so that each pool is composed by the same mice over time. ISs were then retrieved by SLiM-PCR^58^ at different time points from sorted T (CD3+) and B (CD19+) lymphocytes, from myeloid cells (CD11b+) and unsorted blood cells (total MNC). From the DNA purified from all the different sorted samples, we also measured the VCN by ddPCR. Overall, a higher amount of ISs were retrieved from the group of mice transplanted with $Lin^-$ cells transduced with PGK, reflecting the higher level of VCN observed in PGK versus LTR group of transplanted animals. Few statistics on the number of IS collected in each treatment/condition, along with the corresponding VCN, are reported in Table 1. The Shannon entropy index was then computed from each IS sample and the application of a simple spline without shape constraints and without considering any technical confounder yielded the results shown in Fig. 5.

**Figure 6:**
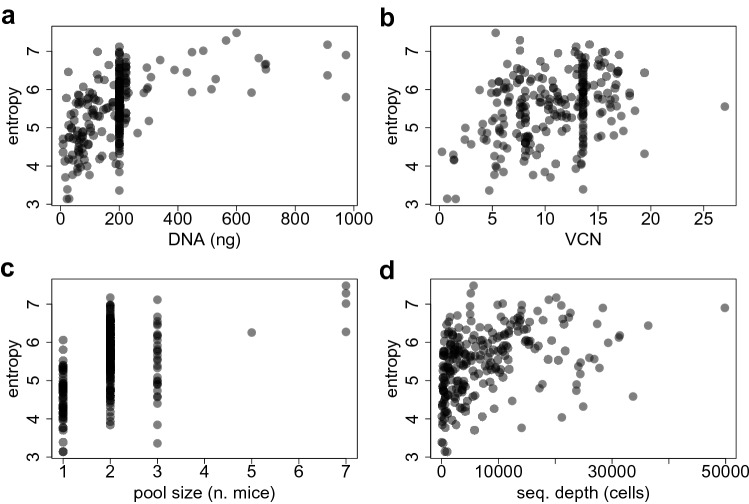
From top left to bottom right: Scatter plot of the raw (unscaled) Shannon entropy index computed from the entire dataset ($n = 242$ IS samples) versus the DNA amount, the vector copy number(VCN), the pool size (PS) and the sequencing depth (SD).

From Fig. 5 we cannot see a clear separation between the entropy profiles of the two vectors PGK and LTR. The prediction intervals overlap so that the differences in the profiles do not appear to be statistically significant. Henceforth, we cannot draw the conclusion that PGK is safer than LTR. However, the high variability of DNA amount (in nanograms), VCN, and the SD used for IS retrieval has a clear impact on the entropy measurements, as suggested by Fig. 6.

Furthermore, since some mice died faster, the size of each pool (PS) decreased over time, leading to variation in the cell counts and in turn in the Shannon diversity index calculations (see Fig. 6). Few statistics of these quantites are reported in Table 1 separately for each vector treatment.

**Table 1:** Mice study: Quartiles and range of the DNA amount, VCN, PS, SD and $n_{IS}$ for the $n = 242$ samples and separately for PGK (left) and LTR (right) treatment conditions.

	DNA	VCN	PS	SD	$n_{IS}$		DNA	DNA	PS	PS	$n_{IS}$
**PGK**						**LTR**					
Min.	8.64	1.31	1.000	60	35.0	Min.	8.64	0.240	1.000	189	35.0
1st Qu.	106.56	10.90	2.000	1969	433.0	1st Qu.	94.50	5.320	1.000	1130	217.0
Median	200.00	13.59	2.000	5881	720.0	Median	200.00	6.300	2.000	2973	383.0
Mean	181.07	12.80	1.964	9351	989.3	Mean	222.88	6.219	2.104	4695	731.9
3rd Qu.	200.25	13.90	2.000	14055	1220.0	3rd Qu.	222.50	7.800	3.000	7390	873.0
Max.	973.00	27.00	3.000	49853	4324.0	Max.	973.00	10.500	7.000	15375	3213.0

This suggests that initial results of Fig. 6 might be biased by the presence of these confounding factors. The heterogeneity of these factors may affect the estimate of the cell counts and in turn the corresponding Shannon entropies. We therefore applied our shape-constrained spline approach of “Methods: shape constrained splines”, including the DNA amount, VCN, SD and PS as potential confounders.

We proceeded as follows: We used the general formulation of Eq. (4), including a shape constrained spline (SCS) term with two interior knots for every confounder, plus a spline term w.r.t. the time decay for every combination of cell lineage/marker (*L*) and viral vector (*V*) as additional factors of interest. In this way we described the entropy decays separately for each combination of cell marker and treatment (viral vector) while removing the bias provided by the potential confounders. We also set the vector specific intercept *V* to zero to make sure all the individuals have the same clonal diversity before the treatments. Therefore, following the general formulation of Eq. (4), the model it has been explicitly defined as

31 $$\log(\mathbf{h}) = \varvec{1}\beta_{0} + \underbrace {{\left[ {\begin{array}{*{20}c} {{\mathbf{C}}_{{dna}} } & {{\mathbf{C}}_{{vcn}} } & {{\mathbf{C}}_{{ps}} } & {{\mathbf{C}}_{{sd}} } \end{array} } \right]}}_{{\mathbf{C}}}\underbrace {{\left[ {\begin{array}{*{20}c} {{\text{ }}\varvec{\beta}_{{dna}} } \\ {\varvec{\beta}_{{vcn}} } \\ {{\text{ }}\varvec{\beta}_{{ps}} } \\ {{\text{ }}\varvec{\beta}_{{sd}} } \end{array} } \right]}}_{{\varvec{\beta}_{c} }} + \underbrace{{\left[ {\begin{array}{*{20}c} {{\mathbb{S}}_{t}^{{l_{1} }} } & {} & {} & {} \\ {} & {\mathbb{S}_{t}^{{l_{2} }} } & {} & {} \\ {} & {} & {{\mathbb{S}}_{t}^{{l_{3} }} } & {} \\ {} & {} & {} & {{\mathbb{S}}_{t}^{{l_{4} }} } \\ \end{array} } \right]}}_{{\mathbf{F}}}\underbrace {{\left[ {\begin{array}{*{20}c} {{\text{ }}\varvec{\beta}_{{l_{1} }} } \\ {\varvec{\beta}_{{l_{2} }} } \\ {\varvec{\beta}_{{l_{3} }} } \\ {{\text{ }}\varvec{\beta}_{{l_{4} }} } \\ \end{array} } \right]}}_{{\varvec{\beta}_{f} }} + \mathbf{\varepsilon}$$

where ${\mathbf {C}}$ binds all the confounder’s SCS bases and $\varvec{\beta }_c$ is the vector with all the corresponding parameters stacked together. Alike, ${\mathbf {F}}$ is a block-diagonal matrix where each block ${\mathbb {S}}_t^l$ is defined as

$$\begin{aligned} {\mathbb {S}}_t^l = \left[ {\begin{matrix} 1 &{} {\mathbb {S}}_t^{l,v_1} &{} \\ 1 &{} &{} {\mathbb {S}}_t^{l, v_2} \\ \end{matrix}} \right] \end{aligned}$$

and each sub-block ${\mathbb {S}}_t^{l, v}$, corresponding to the *l*-th cell lineage and the viral vector *v*, is the basis of a monotone decreasing quadratic spline w.r.t. the time *t* for which we assume a steady-state to the left of the second right boundary knot. Indeed, each mouse pool started with 2-7 mice, which then successively died, until no mouse was left, so that no measurements could be taken anymore and therefore we do not expect any further change in the entropy thereafter. For this purpose we use again the affine transformation defined in Eqs. (17)–(18). Finally, we refer to $\varvec{\beta }_f$ as the vector with all the corresponding parameters stacked together. Therefore in this case the number of confounders is $K = 4$ and the number of additional factors of interest is $J = 8$, namely a spline basis for the time-decay for every combination of the two treatments and the four cell lineages.

**Figure 7:**
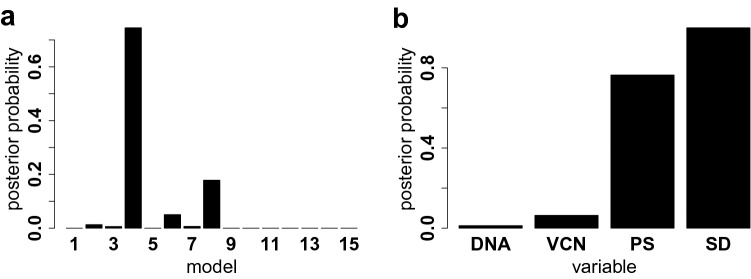
Approximated posterior distribution distribution (left) of the 15 candidate models according to the frequentist model averaging method of Eqs. (24)–(25), and the marginal posterior probabilities of the 4 potential confounders (right).

In order to identify the most important confounders among the candidates, we have fitted our model for each of the $2^4 - 1=15$ possible confounder subsets. Each candidate model included always ${\mathbf {F}}$ as fixed term and featured at least one SCS term in ${\mathbf {C}}$ for the confounders. Then we averaged across the most likely models according to the frequentist criterion defined in Eqs. (24)–(25) and we reported in Fig. 7 the posterior distribution, along with the marginal inclusion probabilities of the four individual confounders.

**Figure 8:**
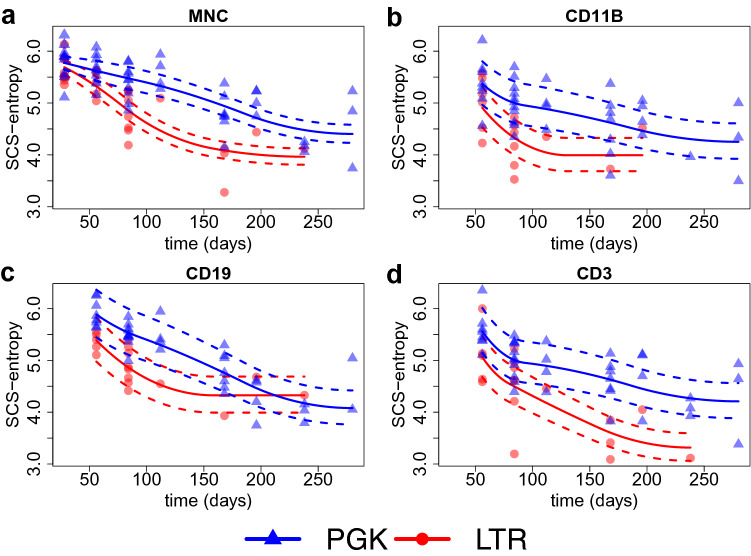
Rescaled Shannon entropies (*y*-axis) over time (*x*-axis) for every combination of cell marker (panels) and viral vector treatment (colors) together with the corresponding SCS-fitted decay splines.

Results from model averaging suggest that the posterior distribution is mainly dominated by three models namely: $\texttt {PS + SD}$ (4th model), $\texttt {VCN + SD}$ (6th model), and $\texttt {SD}$ (8th model). The remaining 12 models get substantially lower posterior probabilities and thus have only negligible effects on the model averaging estimator. Therefore, after computing the frequentist model averaging estimator

32 $$\begin{aligned} \hat{\varvec{\beta}}_{fma} = \begin{bmatrix} {\hat{\beta }}_0&\hat{\varvec{\beta}}_{c,fma}&\hat{\varvec{\beta}}_f \end{bmatrix} ' \end{aligned}$$

of Eq. (24), we used the residuals

33 $$\begin{aligned} \mathbf{h}^{res}&= e^{(\log(\mathbf{h}) - {\mathbf {C}} \hat{\varvec{\beta }}_{c,fma})} \end{aligned}$$

corresponding to the confounder terms as the rescaled values. Rescaled entropies are shown in Fig. 8 together with the lineage$\times$vector-specific spline decays with a confidence interval of 0.95 coverage.

Thanks to the SCS-rescaling approach, a significant difference in the entropy decay for MNC, T-cells (CD3+) and Myeloid cells (CD11b) was observed depending on the genotoxicity level of the vector adopted. Whereas in the B-cell compartment no major differences in the entropy decay under the two vector treatments were observed. Indeed, consistently with the previous results^19,20^, the B-cell compartment is less affected by the genotoxicity of the LTR vector. In Sect. S.4.2 of the supplementary material we show that the RAR and the SRS approaches are less efficient than the proposed SCS approach, cf. Figs. S.5 and S.6 of the supplementary material.

## Discussion

We have shown that the Shannon entropy index, a widely used measure of genetic variability, is strongly affected by the variability of technical factors. We have introduced a shape-constrained splines (SCS) approach aimed to quantitatively measure and remove the effect of confounders from the target of interest. In particular we have shown that our approach can remove confounding effects from the Shannon entropy index. We also have shown that our SCS approach outperforms all the state of the art rarefaction approaches like the RAR^13^ and its ranked-subsampling version^18^. That is, using a correlation test, we have found statistical evidence that the SCS-rescaled diversity measure does not significantly depend on the effect of the confounders anymore. Furthermore, our method is useful for genetic applications, as it provides an unbiased and more affordable measure of clonal diversity, and in turn it avoids to draw misleading results. As an example, the entropy decay of treatment-specific longitudinal studies may be erroneously interpreted if we do not take into account that the changes in the entropy increments may depend more on the confounders than on the biological treatments. Our method allows to discriminate between the two effects and to remove the one that comes from the confounders.

Since our approach is spline-regression based, its main limitation could be related to the available sample size. This is a potential problem when the number *n* of libraries is too low to define a spline basis. For the same reason the degree of the splines and the number of its knots should be chosen carefully. In particular, for the case of only one library/sample it would be possible to rescale its diversity only using the parameters inferred from an external controlled environment, like the VA explored in Sect. 4.1, with a sufficient library size *n*. This is the main price we pay if we switch from a rarefaction-based rescaling approach to a spline-regression based one. Our model averaging approach allows also to rank the impact of the confounders according to their approximated posterior probabilities. We perform model averaging by means of the Bayesian Information Criterion which allows us to get an estimate of the marginal likelihood of each candidate model, and in turn, of the corresponding marginal confounder inclusion posterior probabilities. One possible methodological extension of this framework could be the implementation of a more precise method to estimate the marginal likelihood. This could, for example, either be done by Laplace Integration or by Bayesian thermodynamic integration.

## Supplementary Information

Supplementary Information 1.

## Data Availability

The data that support the findings of this study are openly available in the GitHub repository SHARES at https://github.com/calabrialab/SHARES.

## Code availability

The R code used to produce all the analyses of this work is openly available at the GitHub repository https://github.com/delcore-luca/SCS.

## References

[1] Dunbar CE, High KA, Joung JK, Kohn DB, Ozawa K, Sadelain M (2018). Gene therapy comes of age. Science.

[2] Aiuti A (2013). Lentiviral hematopoietic stem cell gene therapy in patients with Wiskott–Aldrich Syndrome. Science.

[3] Biffi A (2013). Lentiviral hematopoietic stem cell gene therapy benefits Metachromatic Leukodystrophy. Science.

[4] Cesana D (2021). Retrieval of vector integration sites from cell-free DNA. Nat. Med..

[5] Kohn DB (2020). Lentiviral gene therapy for X-linked chronic granulomatous disease. Nat. Med..

[6] Magnani CF, Gaipa G, Lussana F, Belotti D, Gritti G, Napolitano S, Matera G, Cabiati B, Buracchi C, Borleri G (2020). Sleeping Beauty-engineered CAR T cells achieve antileukemic activity without severe toxicities. J. Clin. Investig..

[7] Marktel S, Scaramuzza S, Cicalese MP, Giglio F, Galimberti S, Lidonnici MR, Calbi V, Assanelli A, Bernardo ME, Rossi C (2019). Intrabone hematopoietic stem cell gene therapy for adult and pediatric patients affected by transfusion-dependent ß-thalassemia. Nat. Med..

[8] Scala S, Basso-Ricci L, Dionisio F, Pellin D, Giannelli S, Salerio FA, Leonardelli L, Cicalese MP, Ferrua F, Aiuti A (2018). Dynamics of genetically engineered hematopoietic stem and progenitor cells after autologous transplantation in humans. Nat. Med..

[9] Yuo TS-T, Tseng TA (2021). The environmental product variety and retail rents on central urban shopping areas: A multi-stage spatial data mining method. Environ. Plan. B.

[10] Fu Y (2020). Mutational characterization of hbv reverse transcriptase gene and the genotype-phenotype correlation of antiviral resistance among chinese chronic hepatitis b patients. Emerg. Microbe Infect..

[11] Abina SH-B, Gaspar HB, Blondeau J, Caccavelli L, Charrier S, Buckland K, Picard C, Six E, Himoudi N, Gilmour K (2015). Outcomes following gene therapy in patients with severe Wiskott–Aldrich syndrome. Jama.

[12] McNulty SN, Mann PR, Robinson JA, Duncavage EJ, Pfeifer JD (2020). Impact of reducing DNA input on next-generation sequencing library complexity and variant detection. J. Mol. Diagn..

[13] Sanders HL (1968). Marine benthic diversity: A comparative study. Am. Nat..

[14] Weiss S, Xu ZZ, Peddada S, Amir A, Bittinger K, Gonzalez A, Lozupone C, Zaneveld JR, Vázquez-Baeza Y, Birmingham A, Hyde ER, Knight R (2017). Normalization and microbial differential abundance strategies depend upon data characteristics. Microbiome.

[15] McMurdie PJ, Holmes S (2014). Waste not, want not: Why rarefying microbiome data is inadmissible. PLoS Comput. Biol..

[16] Willis AD (2019). Rarefaction, alpha diversity, and statistics. Front. Microbiol..

[17] Whittaker RH (1972). Evolution and measurement of species diversity. TAXON.

[18] Beule KPL (2020). Improved normalization of species count data in ecology by scaling with ranked subsampling (SRS): Application to microbial communities. PeerJ.

[19] Montini E, Cesana D, Schmidt M, Sanvito F, Ponzoni M, Bartholomae C, Sergi LS, Benedicenti F, Ambrosi A, Di Serio C, Doglioni C, von Kalle C, Naldini L (2006). Hematopoietic stem cell gene transfer in a tumor-prone mouse model uncovers low genotoxicity of lentiviral vector integration. Nat. Biotechnol..

[20] Montini E, Cesana D, Schmidt M, Sanvito F, Bartholomae CC, Ranzani M, Benedicenti F, Sergi LS, Ambrosi A, Ponzoni M, Doglioni C, Serio CD, von Kalle C, Naldini L (2009). The genotoxic potential of retroviral vectors is strongly modulated by vector design and integration site selection in a mouse model of HSC gene therapy. J. Clin. Investig..

[21] Lu R, Neff N, Quake S, Weissman I (2011). Tracking single hematopoietic stem cells in vivo using high-throughput sequencing in conjunction with viral genetic barcoding. Nat. Biotechnol..

[22] Nakamura T, Omasa T (2015). Optimization of cell line development in the GS-CHO expression system using a high-throughput, single cell-based clone selection system. J. Biosci. Bioeng..

[23] Gerrits A, Dykstra B, Kalmykowa OJ, Klauke K, Verovskaya E, Broekhuis MJC, de Haan G, Bystrykh LV (2010). Cellular barcoding tool for clonal analysis in the hematopoietic system. Blood.

[24] Harkey MA (2007). Multiarm high-throughput integration site detection: Limitations of LAM-PCR technology and optimization for clonal analysis. Stem Cells Dev..

[25] Schuster SC (2008). Next-generation sequencing transforms today’s biology. Nat. Methods.

[26] Margulies M, Egholm M, Altman WE, Attiya S, Bader JS, Bemben LA, Berka J, Braverman MS, Chen Y-J, Chen Z, Dewell SB, Du L, Fierro JM, Gomes XV, Godwin BC, He W, Helgesen S, Ho CH, Irzyk GP, Jando SC, Alenquer MLI, Jarvie TP, Jirage KB, Kim J-B, Knight JR, Lanza JR, Leamon JH, Lefkowitz SM, Lei M, Li J, Lohman KL, Lu H, Makhijani VB, McDade KE, McKenna MP, Myers EW, Nickerson E, Nobile JR, Plant R, Puc BP, Ronan MT, Roth GT, Sarkis GJ, Simons JF, Simpson JW, Srinivasan M, Tartaro KR, Tomasz A, Vogt KA, Volkmer GA, Wang SH, Wang Y, Weiner MP, Yu P, Begley RF, Rothberg JM (2005). Genome sequencing in microfabricated high-density picolitre reactors. Nature.

[27] Demkow U, Ploski R (2015). Clinical Applications for Next-Generation Sequencing.

[28] Shendure J, Mitra RD, Varma C, Church GM (2004). Advanced sequencing technologies: Methods and goals. Nat. Rev. Genet..

[29] Ledergerber C, Dessimoz C (2011). Base-calling for next-generation sequencing platforms. Brief. Bioinform..

[30] Chang F, Li MM (2013). Clinical application of amplicon-based next-generation sequencing in cancer. Cancer Genet..

[31] Kohlmann A, Grossmann V, Haferlach T (2012). Integration of Next-Generation Sequencing into clinical practice: Are we there yet?. Semin. Oncol..

[32] Gargis AS, Kalman L, Bick DP, da Silva C, Dimmock DP, Funke BH, Gowrisankar S, Hegde MR, Kulkarni S, Mason CE, Nagarajan R, Voelkerding KV, Worthey EA, Aziz N, Barnes J, Bennett SF, Bisht H, Church DM, Dimitrova Z, Gargis SR, Hafez N, Hambuch T, Hyland FCL, Luna RA, MacCannell D, Mann T, McCluskey MR, McDaniel TK, Ganova-Raeva LM, Rehm HL, Reid J, Campo DS, Resnick RB, Ridge PG, Salit ML, Skums P, Wong L-JC, Zehnbauer BA, Zook JM, Lubin IM (2015). Good laboratory practice for clinical next-generation sequencing informatics pipelines. Nat. Biotechnol..

[33] Biasco L, Pellin D, Scala S, Dionisio F, Basso-Ricci L, Leonardelli L, Scaramuzza S, Baricordi C, Ferrua F, Cicalese M, Giannelli S, Neduva V, Dow D, Schmidt M, Von Kalle C, Roncarolo M, Ciceri F, Vicard P, Wit E, Di Serio C, Naldini L, Aiuti A (2016). In vivo tracking of human hematopoiesis reveals patterns of clonal dynamics during early and steady-state reconstitution phases. Cell Stem Cell.

[34] Wu C, Li B, Lu R, Koelle S, Yang Y, Jares A, Krouse A, Metzger M, Liang F, Loré K, Wu C, Donahue R, Chen I, Weissman I, Dunbar C (2014). Clonal tracking of rhesus macaque hematopoiesis highlights a distinct lineage origin for natural killer cells. Cell Stem Cell.

[35] Mazurier F, Gan OI, McKenzie JL, Doedens M, Dick JE (2004). Lentivector-mediated clonal tracking reveals intrinsic heterogeneity in the human hematopoietic stem cell compartment and culture-induced stem cell impairment. Blood.

[36] Biasco L, Rothe M, Schott JW, Schambach A (2020). Integrating vectors for gene therapy and clonal tracking of engineered hematopoiesis. Hematology.

[37] Shannon CE (1948). A mathematical theory of communication. Bell Syst. Tech. J..

[38] Kullback S, Leibler RA (1951). On information and sufficiency. Ann. Math. Stat..

[39] Carboni I, Fattorini P, Previderè C, Ciglieri SS, Iozzi S, Nutini A, Contini E, Pescucci C, Torricelli F, Ricci U (2015). Evaluation of the reliability of the data generated by next generation sequencing from artificially degraded DNA samples. Forensic Sci. Int..

[40] Peng Y, Leung HCM, Yiu SM, Chin FYL (2012). IDBA-UD: A de novo assembler for single-cell and metagenomic sequencing data with highly uneven depth. Bioinformatics.

[41] Pereira-Marques J, Hout A, Ferreira RM, Weber M, Pinto-Ribeiro I, van Doorn L-J, Knetsch CW, Figueiredo C (2019). Impact of host DNA and sequencing depth on the taxonomic resolution of whole metagenome sequencing for microbiome analysis. Front. Microbiol..

[42] Hahn A, Sanyal A, Perez GF, Colberg-Poley AM, Campos J, Rose MC, Pérez-Losada M (2016). Different next generation sequencing platforms produce different microbial profiles and diversity in cystic fibrosis sputum. J. Microbiol. Methods.

[43] Sabina J, Leamon JH (2015). Bias in Whole Genome Amplification: Causes and Considerations.

[44] Kozich JJ, Westcott SL, Baxter NT, Highlander SK, Schloss PD (2013). Development of a dual-index sequencing strategy and curation pipeline for analyzing amplicon sequence data on the MiSeq illumina sequencing platform. Appl. Environ. Microbiol..

[45] Nakayama Y, Yamaguchi H, Einaga N, Esumi M (2016). Pitfalls of DNA quantification using DNA-binding fluorescent dyes and suggested solutions. PLoS ONE.

[46] Robin JD, Ludlow AT, LaRanger R, Wright WE, Shay JW (2016). Comparison of DNA quantification methods for next generation sequencing. Sci. Rep..

[47] Pya N, Wood SN (2015). Shape constrained additive models. Stat. Comput..

[48] Bollaerts K, Eilers PH, Van Mechelen I (2006). Simple and multiple P-splines regression with shape constraints. Br. J. Math. Stat. Psychol..

[49] Brezger A, Steiner WJ (2008). Monotonic regression based on bayesian p-splines: An application to estimating price response functions from store-level scanner data. J. Bus. Econ. Stat..

[50] Fritsch FN, Carlson RE (1980). Monotone piecewise cubic interpolation. SIAM J. Numer. Anal..

[51] Meyer MC (2008). Inference using shape-restricted regression splines. Ann. Appl. Stat..

[52] Meyer MC (2018). A framework for estimation and inference in generalized additive models with shape and order restrictions. Stat. Sci..

[53] De Boor C, De Boor C, Mathématicien E-U, De Boor C, De Boor C (1978). A Practical Guide to Splines.

[54] Liao X, Meyer MC (2014). coneproj: An R package for the primal or dual cone projections with routines for constrained regression. J. Stat. Softw..

[55] Burnham KP, Anderson DR, Huyvaert KP (2011). AIC model selection and multimodel inference in behavioral ecology: Some background, observations, and comparisons. Behav. Ecol. Sociobiol..

[56] Spinozzi G, Calabria A, Brasca S, Beretta S, Merelli I, Milanesi L, Montini E (2017). VISPA2: A scalable pipeline for high-throughput identification and annotation of vector integration sites. BMC Bioinform..

[57] Berry CC, Gillet NA, Melamed A, Gormley N, Bangham CRM, Bushman FD (2012). Estimating abundances of retroviral insertion sites from DNA fragment length data. Bioinformatics.

[58] Benedicenti F, Calabria A, Cesana D, Albertini A, Tenderini E, Spinozzi G, Neduva V, Richard A, Brugman M, Dow D (2019). Sonication linker mediated-PCR (SLiM-PCR), an efficient method for quantitative retrieval of vector integration sites. Hum.Gene Ther..

