# Supplementary material for: Normalization of clonal diversity in gene therapy studies using shape constrained splines
Source: Sci Rep. 2022 Mar 9;12:3836. doi: 10.1038/s41598-022-05837-0 (PMC8907296; doi:10.1038/s41598-022-05837-0)
Supplement: Supplementary file 1 — Supplementary Information 1. [file 41598_2022_5837_MOESM1_ESM.pdf]

Supplementary Material for the paper "Normalization of clonal diversity in gene therapy studies using shape constrained splines"

L. Del Core et al.

S.1 Protocols of IS retrieval: SLiM-PCR

The retrieval of integration sites mimics the procedure described in [1] referred as Sonication Linker Mediated (SLiM-) PCR. In this method, the genomic DNA of vector marked cells is sonicated to obtain randomly sheared fragments, ligated to a synthetic DNA linker cassette needed as template for the next PCR amplification. The tagged DNA is then used as template for PCR using oligonucleotides, complementary to both vector sequences and linker cassette, in order to amplify the specific vector/cell genome junctions contained in-between. Since the (random) DNA fragmentation, due to sonication, occurs before PCR amplification, a clonal population harbouring the same IS will produce a number of DNA fragments containing the vector/cell genome junctions of different sizes that will be proportional to the initial number of contributing cells.

S.2 In-vitro Assay

In this experimental setup the genomic DNA of a bulk-transduced cell line harboring lentiviral insertions randomly distributed in the genome. This is a controlled environment designed to quantify the impact of several confounding factors to the clonal entropy. More specifically, the genomic DNA is obtained from a polyclonal lentiviral-vector (LV) marked cell line (JY). The DNA material was sheared by sonication, end-repaired and adenylated, split in technical triplicates, ligated of the barcoded linker cassettes and subjected to PCR amplification protocols for IS retrieval. Clonal quantification is obtained using the R package SonicLength[2]. The total amount of integrations found in a sample, namely the total number of sample's sequencing reads, has been used as proxy for the sample's sequencing depth (SD). Few summary statistics of the DNA amount, multiplicity of infection (MOI), VCN, number of distinct IS (n_{IS}) and SD are provided in Table S.1. In Table S.2 we provide the VCN, the total number of distinct ISs and the total SD in each condition, that is for every combination of DNA amount and MOI. The sample-specific information is provided in Table S.3.

DNA	MOI	VCN	n_{IS}	SD
Min. : 5.00	Min. : 0.1	Min. : 0.484	Min. : 22	Min. : 41
1st Qu.: 5.00	1st Qu.: 0.1	1st Qu.: 0.484	1st Qu.: 188	1st Qu.: 419
Median : 20.00	Median : 1.0	Median : 2.850	Median : 1004	Median : 1340
Mean : 41.67	Mean : 3.7	Mean : 7.478	Mean : 5544	Mean : 6032
3rd Qu.:100.00	3rd Qu.:10.0	3rd Qu.:19.100	3rd Qu.: 3811	3rd Qu.: 4398
Max. :100.00	Max. :10.0	Max. :19.100	Max. :40575	Max. :41787

Table S.1: **In-vitro assay (VA)**: Quartiles and ranges of the DNA amount, MOI, VCN, n_{IS} and SD for the $n = 27$ samples.

	DNA	MOI	VCN	n_{IS}	SD
1	5	0.10	0.48	143.00	361.00
2	20	0.10	0.48	548.00	1288.00
3	100	0.10	0.48	3307.00	4936.00
4	5	1.00	2.85	578.00	1043.00
5	20	1.00	2.85	2008.00	3044.00
6	100	1.00	2.85	19239.00	22021.00
7	5	10.00	19.10	3166.00	4163.00
8	20	10.00	19.10	10506.00	12331.00
9	100	10.00	19.10	110182.00	113689.00

Table S.2: **In-vitro assay (VA)**: VCN, total number of distinct ISs and total SD in each of the nine conditions (combination of DNA amount and MOI).

S.3 Mice study

S.3.1 Ethics oversight

- Wild-type C57BL6/J mice were obtained from Charles River (Charles River Laboratories). $C57BL6/J$-$Cdkn2a^{-/-}$ mice were obtained from the National Cancer Institute Mouse Models of Human Cancer Consortium. All mice were bred and kept in the dedicated pathogen-free animal facility at the “Dibit2 stabulario”.

- All procedures were performed according to protocols approved by the Animal Care and Use Committee of the San Raffaele Institute (IACUC 619) and communicated to the Ministry of Health and local authorities according to Italian law.

- All experiments were performed in accordance with relevant guidelines and regulations.

- The reporting in the manuscript follows the recommendations in the ARRIVE guidelines.

DNA	MOI	VCN	n_{IS}	SD
100	0.10	0.48	1300	1954.00
100	1.00	2.85	6082	7264.00
100	10.00	19.10	40575	41787.00
20	0.10	0.48	183	429.00
20	1.00	2.85	649	1087.00
20	10.00	19.10	3933	4631.00
5	0.10	0.48	78	250.00
5	1.00	2.85	204	395.00
5	10.00	19.10	1051	1492.00
100	0.10	0.48	1074	1557.00
100	1.00	2.85	6632	7479.00
100	10.00	19.10	34629	35743.00
20	0.10	0.48	177	450.00
20	1.00	2.85	692	966.00
20	10.00	19.10	2884	3534.00
5	0.10	0.48	43	70.00
5	1.00	2.85	188	387.00
5	10.00	19.10	1004	1340.00
100	0.10	0.48	933	1425.00
100	1.00	2.85	6525	7278.00
100	10.00	19.10	34978	36159.00
20	0.10	0.48	188	409.00
20	1.00	2.85	667	991.00
20	10.00	19.10	3689	4166.00
5	0.10	0.48	22	41.00
5	1.00	2.85	186	261.00
5	10.00	19.10	1111	1331.00

Table S.3: **In-vitro assay (VA)**: VCN, number of distinct ISs and SD in each of the $n = 27$ samples.

S.3.2 Vector production

All the concentrated LV stocks were pseudotyped with the VSV.G envelope and were produced by transient co-transfection of 4 plasmids in 293T cells and tittered on HeLa cells as described in [3].

S.3.3 Isolation and transduction of hematopoietic progenitors

Eight-week-old $Cdkn2a^{-/-}$ mice were sacrified by CO2 inhalation, and BM was harvested by flushing femurs and tibiae with PBS-2% FBS (FBS; Invitrogen). Lin^{-} cells were purified by lineage-marker' negative selection using the Enrichment of Murine Hematopoietic Progenitors Kit (StemCell Technologies), plated at a density of 1×10^6 cells/ml, and cultured in StemSpan SFEM expansionmedium (StemCell Technologies) with a cytokine cocktail composed of 100 ng/ml SCF, 50 ng/ml thrombopoietin, 100 ng/ml Flt3 lig-

and, and 20 ng/ml IL-3 (PeproTech). After 2 hours pre-stimulation, Lin^- cells were split and subjected to mock or LV transduction (108 TU/ml, MOI 100). After 18 hours, cells were washed and transplanted into lethally irradiated witd-type animals. A sample of cells was kept in culture for 10 days to assess GFP expression by FACS analysis

S.3.4 HSPCs Transduction

After 2 hours of prestimulation, Lin^- cells were subjected to Mock, either with SIN.LV.PGK.GFP.PRE or LV.SF.LTR transduction ($MOI = 100$).
Vector titers were:
- SIN.LV.PGK.GFP.PRE 3×10^9 TU/ml and 5.5×10^9 TU/ml
- LV.SF.LTR 3×10^9 TU/ml and 1.7×10^9 TU/ml.

Cells were kept in culture for 24 hours before transplant. In all the experiments, a sample of cells was kept for 14 days in culture in StemSpam complemented as above described diluted in a 1:2 ratio with RPMI medium 10% FBS, Penicillin and Streptomycin (100U/ml each) and L-glutamine 2mM, to assess GFP expression by FACS analysis and for genomic DNA extraction procedures.

S.3.5 HSPCs transplantation in Wild-type C57BL6/J mice

Seven weeks-old wild type female C57BL6/J mice were lethally irradiated with 7.5 Gy administered in two sub-lethal doses 2 hour apart. 24 hours after irradiation, mice were heated under an infrared heat lamp and transplanted with vector- and mock-transduced cells (5-7.5 $\times 10^5$ cells/mouse in 200μl of PBS) by intravenous tail vein injection. Recipient mice were kept under prophylactic antibiotic treatment starting 1 week prior to transplantation until 2 weeks post-transplant. The engraftment of transduced cells was evaluated at 8 week post-transplantation by flow cytometry by quantifying the overall percentage of GFP-expressing cells. Untransduced cells were used as negative control to set parameters and gates.

S.3.6 Serial blood sampling from transplanted mice

200 μl of peripheral blood were taken from all transplanted mice via retro-orbital bleeding using heparinized capillary glass tubes. Bleedings were performed approximately every 4 weeks starting 4 weeks after transplantation. Prior to all bleeding procedures mice were anesthetized via isoflurane inhalation.

S.3.7 Isolation of CD3+, CD11b+ and CD19+ cells from total peripheral blood (PB)

Bleedings were performed approximately every 4 weeks starting 4 weeks after transplantation. Prior to bleeding procedures mice were anesthetized via isoflurane inhalation. $100\mu l$ of peripheral blood were collected from transplanted mice via retro-orbital bleeding using heparinized capillary glass tubes. B-lymphocytes, T- lymphocytes and myeloid cells were isolated from total peripheral blood by sorting of cells using Lineage-specific antibodies (Abs) against murine CD11b (myeloid marker), CD19 (B-lymphoid marker), CD3 (T-lymphoid marker). Antibodies used were purchased from BD Biosciences Pharmingen: rat anti-mouse CD11b (BV421); rat anti-mouse CD3 (PE); rat anti-mouse CD19 (APC). Sorted cells and the remaining total peripheral blood cells were pelleted and stored at $-80°C$ in a residual volume of 10-$30\mu l$ of PBS 2% FBS.

S.3.8 Genomic extraction from sorted samples and VCN

Sorted cells and total peripheral blood cells were thawed and genomic DNA was purified using the QIAamp DNA Micro Kit from Qiagen (56304), according to manufacturer's instructions. Because of the low amount of starting material, carrier RNA (provided with the kit) was added to Buffer AL to enhance binding of DNA to the column membrane, and final incubation with Buffer AE was extended to 5 minutes, in order to increase DNA yield, as recommended by the manufacturer. DNA was eluted in $35\mu l$ of Buffer AE. Genomic DNA Concentration was assessed using the Qubit dsDNA HS Assay with the Qubit Fluorometer (ThermoFisher Scientific). Vector copy number was determined by droplet-digital PCR, using primer and probe system using probes complementary to mouse genomic B-actin and the common psi-signal region of LV described in [4]. VCN was determined as the ratio between the amounts of LV versus total DNA evaluated by B-actin that was specific for LV sequences and the β-actin murine gene. Each sample was run in duplicate in a total volume of $20\mu l$ reaction.

S.4 Supplementary figures

S.4.1 Fitting quadratic and cubic splines

We fitted and compared quadratic and cubic splines on the VA data of Section "In-vitro assay" of the main paper. First, we used quadratic splines with one interior knot for the DNA amount and the VCN and two interior knots for the sequencing depth (like in the paper). Second, we used cubic splines with no interior knots for the DNA amount and the VCN and two interior knots for the sequencing depth. The number of knots were chosen, so as to get a full-rank design matrix, and in turn, a positive-definite quadratic

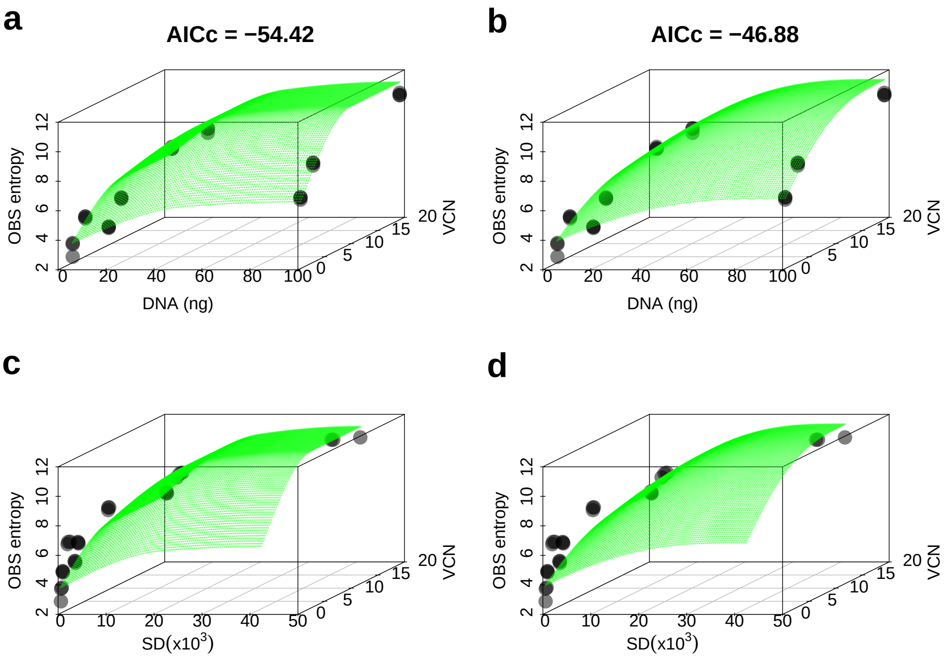

Figure S.1: In-vitro Assay (VA): Quadratic (left) and cubic (right) spline fitting of the shannon entropies (z-axis) against the candidate confounders. The x-axis refers to the DNA amount (top panels) or the SD (bottom panels). The y-axis refers to the VCN. The corresponding AIC is reported at the top.

form, which is needed during optimization. Results are shown below in figure S.1. The results in figure S.1 show that there is not much difference between the two fitted surfaces (visual inspection). To be more objective, we also computed the two corrected Akaike Information criteria (cAIC) and found that the quadratic spline yields a lower cAIC value than the cubic spline.

S.4.2 Comparisons of rescaling methods

To make clearer what we mean by 'RAR and SCS leave significant rank correlations (=dependencies)' in Section "In-vitro assay" of the main paper, we include here a couple of additional figures. Figure S.2 shows an additional graphical representation of the Spearman's rank correlation tests performed in Section "In-vitro assay" of the main paper. Figure S.2 clearly shows that our SCS-rescaled entropies outperform both RAR- and SRS- rescaled entropies in terms of dependence on the candidate confounders. Indeed, the red-highlighted square indicate that both RAR- and SRS- rescaled entropies still suffers from significant dependence on the confounders. Whereas, the

SCS-rescaled entropies do not depend on any of the confounders anymore (all three correlations are insignificant). These are the results that we report in figure 3. Figure S.3 shows the observed entropies and the SCS-, RAR-, SRS-rescaled Shannon entropies against the candidate confounders, along with the fitted surfaces. This figure shows that only the SCS-rescaled entropies do not depend on the confounders anymore, as the fitted surface is an (almost) horizontal plane. The surfaces fitted for RAR- and SRS- rescaled entropies are non-horizontal planes, as the entropies still depend on the confounders.

These analyses better clarify why SCS performs significantly better than the competitor methods. For the mice study data we do not know the 'ground truth', so that we cannot objectively cross-compare the performances of the different methods. However, we have applied all three methods to monitor the rescaled entropies in the mice study. The results are reported in figures S.4 - S.6, showing that the proposed SCS-rescaling method yields the tightest confidence intervals and shows the clearest (smoothest) trends.

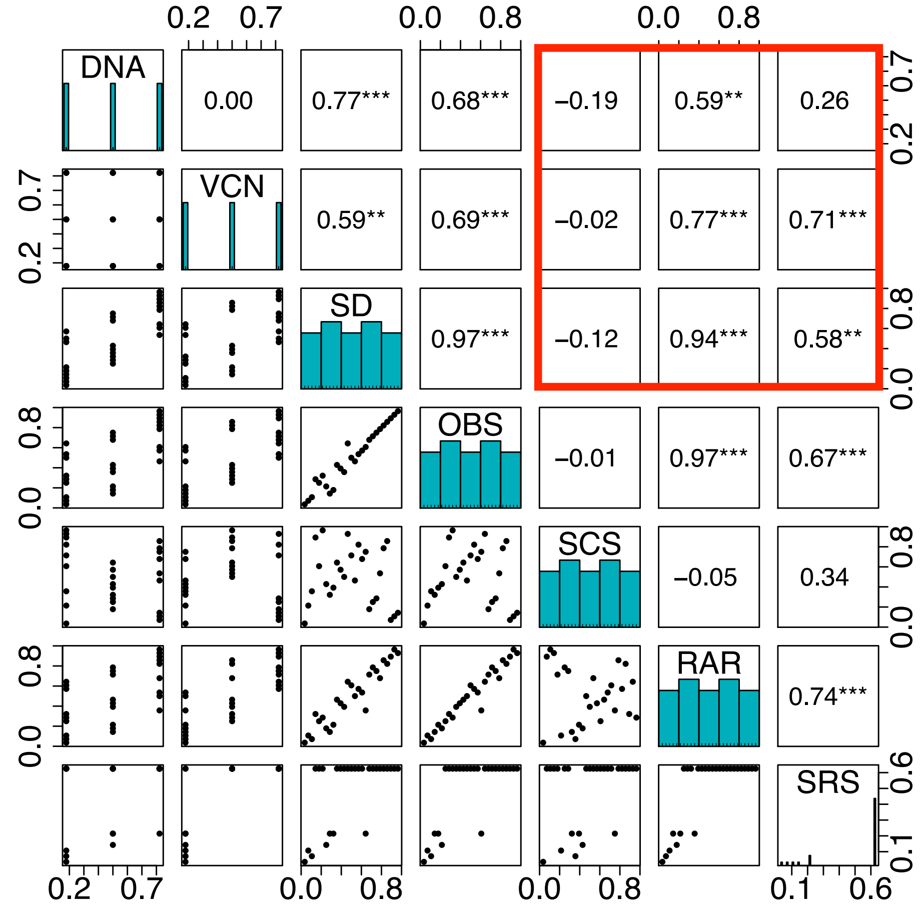

Figure S.2: Pairwise correlation scatter plots between ranks of the candidate confounders (DNA, VCN, SD), the ranks of the observed entropies (OBS), and the ranks of the rescaled entropies obtained with the three methods SCS, RAR and SRS. The ranks have been rescaled by the sample size. Significant correlations are labelled with star symbols "*", where the significance level increases (p-values decreases) in the number of stars. **We have put a red-highlighted square around the most relevant correlations, which we have also displayed in the histograms of figure 3.**

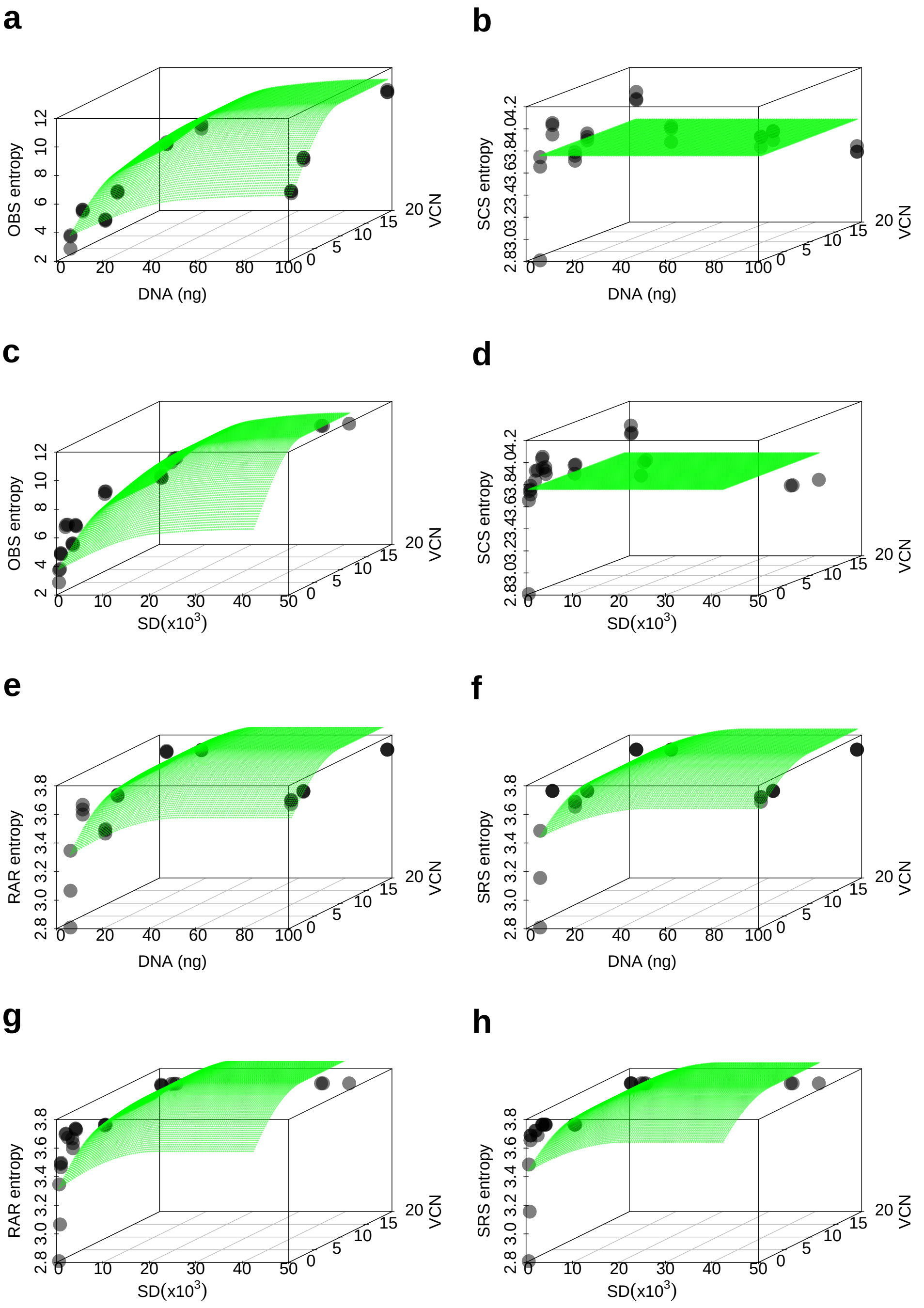

Figure S.3: Each panel shows a three-dimensional scatterplot. The x-axes refer to the DNA amount or the SD. The y-axes refer to the VCN, and the z-axes refer to the entropies. The eight panels refer to (a,c): the observed entropies, (b,d): the SCS-rescaled entropies, (e,g): the RAR-rescaled entropies, and (f,h): the SRS-rescaled entropies. In each panel the fitted surfaces are shown in green. We note that panels a-d correspond to Figure 2 of the main paper.

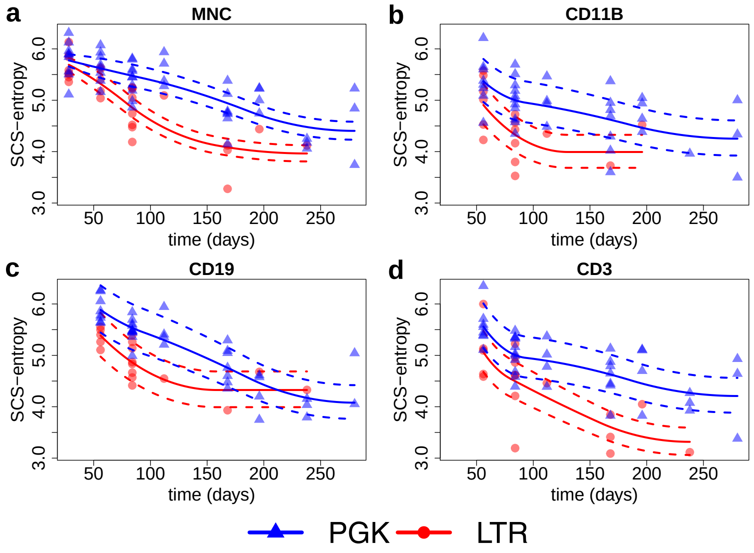

Figure S.4: **SCS-rescaled entropies**: For each of the four lineages there is a panel with two fitted curves for the two viral vector conditions (different colors). The dotted lines refer to confidence intervals with .95 coverage. We note that this figure is a reproduction of Figure 8 of the main paper.

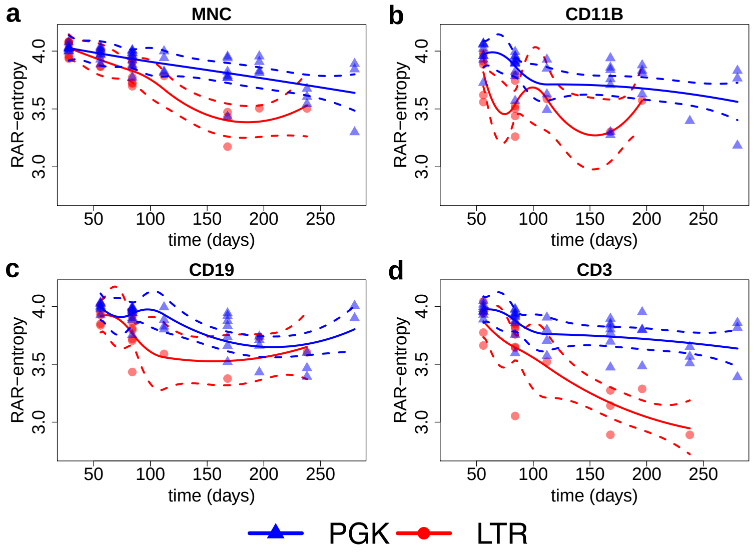

Figure S.5: **RAR-rescaled entropies**: For each of the four lineages there is a panel with two fitted curves for the two viral vector conditions (different colors). The dotted lines refer to confidence intervals with .95 coverage.

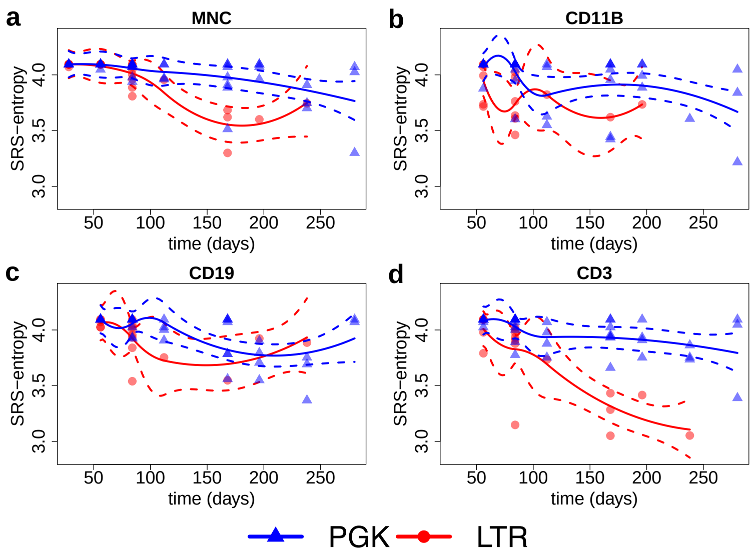

Figure S.6: **SRS-rescaled entropies**: For each of the four lineages there is a panel with two fitted curves for the two viral vector conditions (different colors). The dotted lines refer to confidence intervals with .95 coverage.

References

[1] F. Benedicenti, A. Calabria, D. Cesana, A. Albertini, E. Tenderini, G. Spinozzi, V. Neduva, A. Richard, M. Brugman, D. Dow, *et al.*, "Sonication linker mediated-PCR (SLiM-PCR), an efficient method for quantitative retrieval of vector integration sites," in *Human Gene Therapy*, vol. 30, pp. A214–A215, 2019.

[2] C. C. Berry, N. A. Gillet, A. Melamed, N. Gormley, C. R. M. Bangham, and F. D. Bushman, "Estimating abundances of retroviral insertion sites from DNA fragment length data," *Bioinformatics*, vol. 28, pp. 755–762, 03 2012.

[3] E. Montini, D. Cesana, M. Schmidt, F. Sanvito, M. Ponzoni, C. Bartholomae, L. S. Sergi, F. Benedicenti, A. Ambrosi, C. Di Serio, C. Doglioni, C. von Kalle, and L. Naldini, "Hematopoietic stem cell gene transfer in a tumor-prone mouse model uncovers low genotoxicity of lentiviral vector integration," *Nature Biotechnology*, vol. 24, no. 6, pp. 687–696, 2006.

[4] E. Montini, D. Cesana, M. Schmidt, F. Sanvito, C. C. Bartholomae, M. Ranzani, F. Benedicenti, L. S. Sergi, A. Ambrosi, M. Ponzoni, C. Doglioni, C. D. Serio, C. von Kalle, and L. Naldini, "The genotoxic potential of retroviral vectors is strongly modulated by vector design and integration site selection in a mouse model of HSC gene therapy," *The Journal of Clinical Investigation*, vol. 119, no. 4, pp. 964–975, 2009.
